# Transfer of labile organic matter and microbes from the ocean surface to the marine aerosol: an experimental approach

**DOI:** 10.1038/s41598-017-10563-z

**Published:** 2017-09-13

**Authors:** Eugenio Rastelli, Cinzia Corinaldesi, Antonio Dell’Anno, Marco Lo Martire, Silvestro Greco, Maria Cristina Facchini, Matteo Rinaldi, Colin O’Dowd, Darius Ceburnis, Roberto Danovaro

**Affiliations:** 1 Department of Life and Environmental Sciences, Polytechnic University of Marche, Ancona, Italy; 2 Stazione Zoologica Anton Dohrn, Villa Comunale, Naples, Italy; 3 Department of Sciences and Engineering of Materials, Environment and Urbanistics, Polytechnic University of Marche, Ancona, Italy; 4 Istituto Superiore per la Ricerca Ambientale, ISPRA, Roma, Italy; 5 Institute of Atmospheric Sciences and Climate (ISAC), National Research Council (CNR), Bologna, Italy; 6 School of Physics & Centre for Climate and Air Pollution Studies, Ryan Institute, National University of Ireland, Galway Galway, Ireland

## Abstract

Surface ocean bubble-bursting generates aerosols composed of microscopic salt-water droplets, enriched in marine organic matter. The organic fraction profoundly influences aerosols’ properties, by scattering solar radiations and nucleating water particles. Still little is known on the biochemical and microbiological composition of these organic particles. In the present study, we experimentally simulated the bursting of bubbles at the seawater surface of the North-Eastern Atlantic Ocean, analysing the organic materials and the diversity of the bacteria in the source-seawaters and in the produced aerosols. We show that, compared with seawater, the sub-micron aerosol particles were highly enriched in organic matter (up to 140,000x for lipids, 120,000x for proteins and 100,000x for carbohydrates). Also DNA, viruses and prokaryotes were significantly enriched (up to 30,000, 250 and 45x, respectively). The relative importance of the organic components in the aerosol did not reflect those in the seawater, suggesting their selective transfer. Molecular analyses indicate the presence of selective transfers also for bacterial genotypes, highlighting higher contribution of less abundant seawater bacterial taxa to the marine aerosol. Overall, our results open new perspectives in the study of microbial dispersal through marine aerosol and provide new insights for a better understanding of climate-regulating processes of global relevance.

## Introduction

Marine aerosol is of primary importance for atmospheric processes from local to global scale. It influences the radiation balance of the earth, scattering and absorbing solar radiations either directly or by cloud-condensation nuclei (CCN) and ice-nucleating particles (INPs) formation^1–3^. Consequently, changes in marine aerosol abundance and/or composition can significantly influence the global climate^4–7^. Marine aerosol is composed of diverse inorganic species, but very complex mixtures of organic compounds also contribute to its overall mass^5, 6^. The quantification and characterisation of these organic materials in marine aerosol is at present a largely unexplored issue and a technical challenge, with available information still very scant^8–11^. Recent evidences suggest that marine primary aerosols produced by wind-induced bubble bursting in the ocean^12^ can be highly enriched in organic matter^5, 11, 13–15^ and microorganisms^16–19^, including prokaryotes and viruses^20–22^.

Microbes in the surface oceans are intermediary sources or sinks for inorganic and organic compounds. Surface marine waters typically display higher microbial abundances and concentrations of dissolved and particulate organic matter than in sub-surface waters (up to 3-orders of magnitude)^11, 20, 23^. This material is cycled/transferred through the food web, and microbes can significantly influence the exchange of organic compounds across the air/water interface and hence the production and composition of marine aerosols^3, 20, 24–27^. However, to date attempts to explore the relative transfer of bacteria, viruses and different biopolymeric (i.e., labile, more reactive) organic compounds from the surface seawater to the aerosol is still scarce^5, 10, 28^.

In this study, we conducted bubble-bursting experiments simulating the formation of primary airborne marine aerosols from surface seawater samples collected in the North-Eastern Atlantic Ocean, over a period of two weeks. To provide new insights on the sea-to-air transfer of different organic particles and compounds, we adapted and optimized existing analytical tools to quantify the main biochemical classes of organic compounds (proteins, carbohydrates, lipids), viruses and prokaryotes in marine aerosol samples. Moreover, we quantified for the first time the transfer of extracellular DNA from the ocean surface layer to the aerosol. The diversity of bacterial genotypes was investigated through molecular fingerprinting of seawater samples and of the contextual produced aerosols, to provide new insights on the potential dispersal of bacteria via aerosol and the contribution of different bacterial taxa to the generation of marine aerosols.

## Results and Discussion

The quantity and quality of the organic fraction of marine aerosols determine their potential to influence Earth climate, with relevance on the functioning of the ecosystems at global scale^3, 5, 29^. Despite this, the transfer of organics and microorganisms from the sea surface to the atmosphere currently remain poorly investigated when compared to harmful compounds and viral/bacterial pathogens transported through aerosols in urban and rural areas, hospitals or workplaces^22, 30–32^.

**Figure 1:**
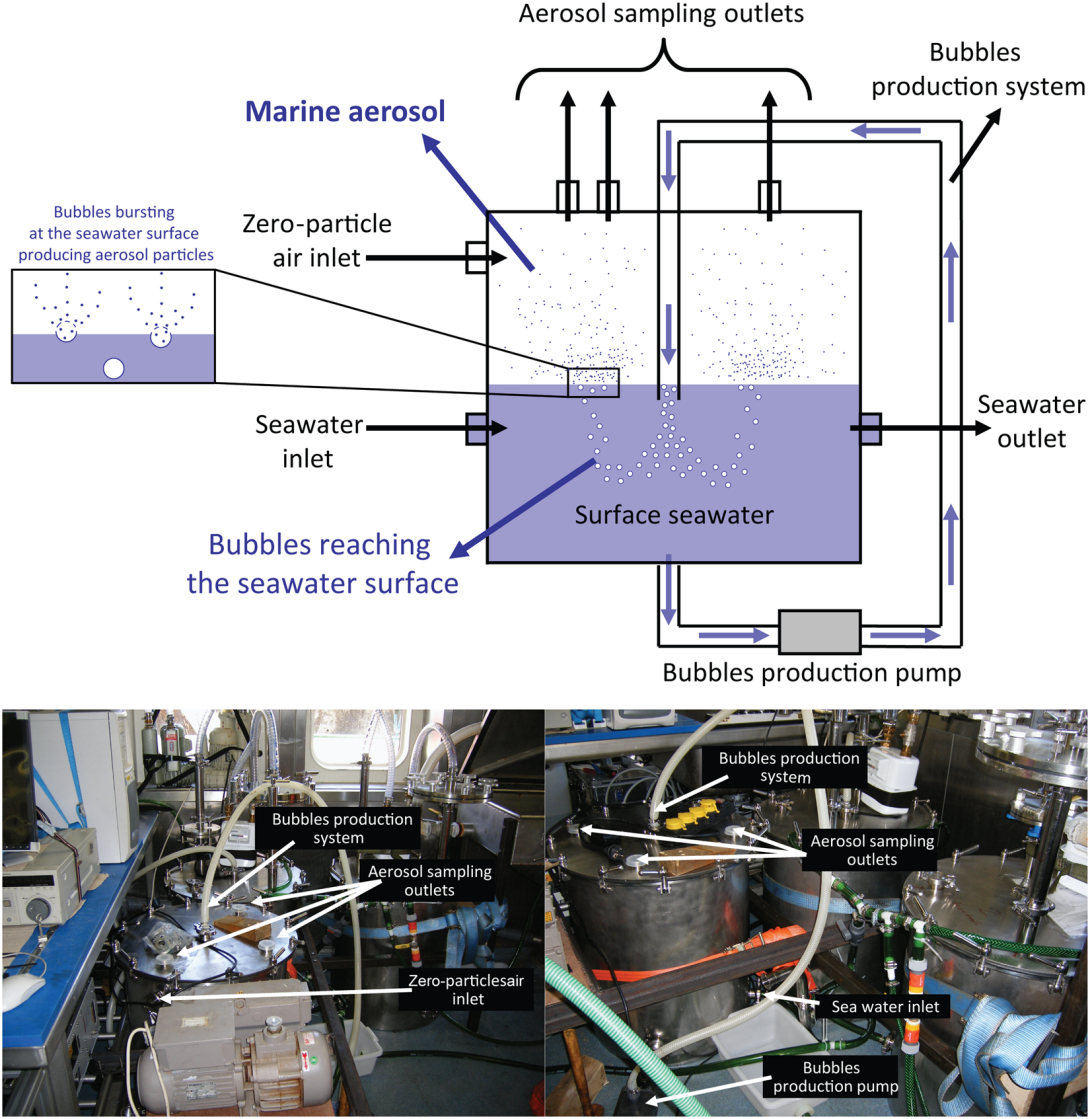
Experimental system for the production of marine aerosol. The upper scheme illustrates the functioning of the bubble-bursting experimental systems. The lower images show details of the technical instrumentation utilised for the bubble-bursting experiments onboard R/V Celtic Explorer to produce and collect marine aerosol samples and aliquots of the corresponding source-seawater used for their generation. Additional details on the experimental setup can be found in ref. 14.

**Figure 2:**
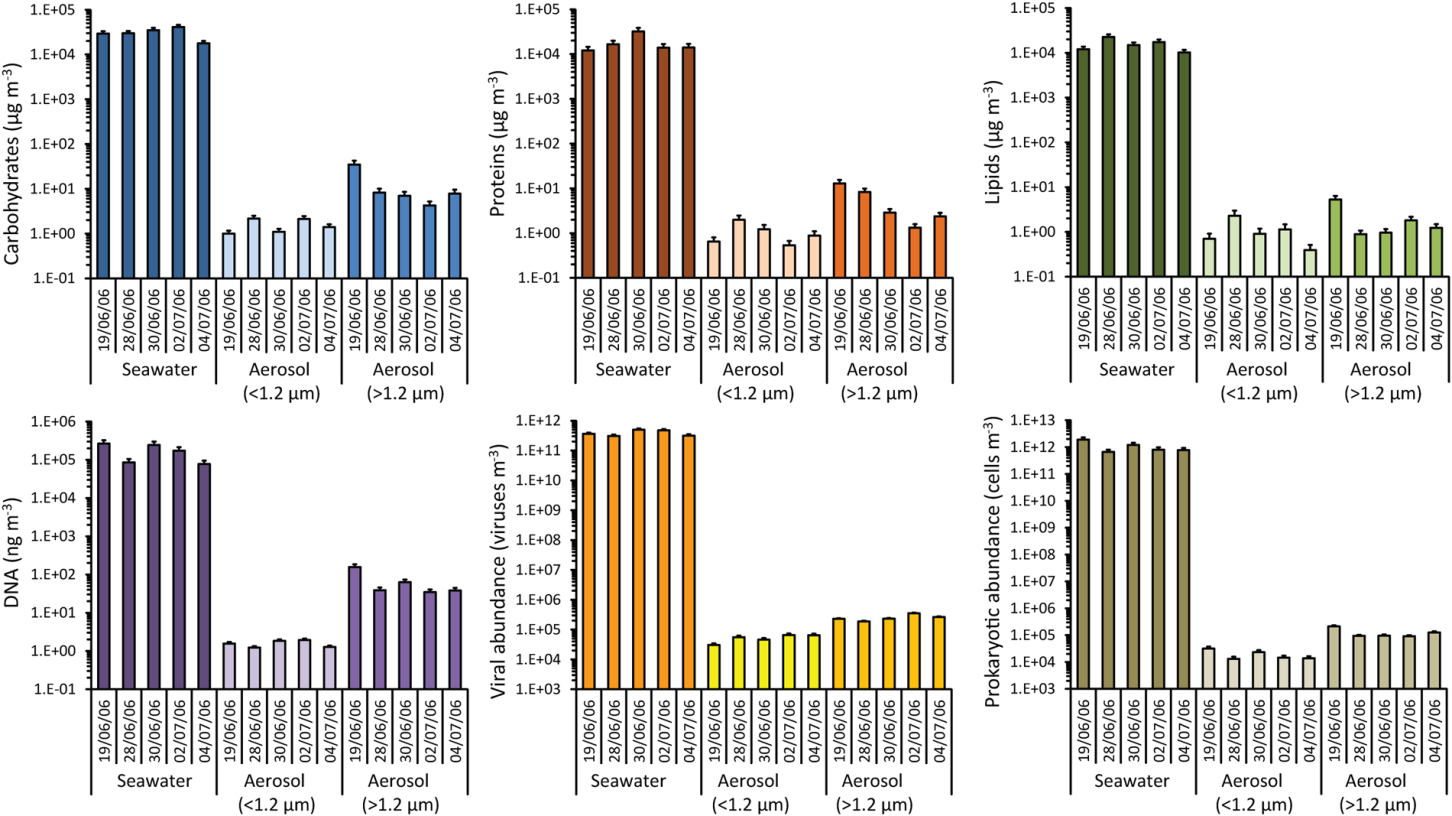
Organic matter and microbes in surface seawater and aerosol samples. The panel reports the concentrations of the different organic compounds analysed in the present study (carbohydrates, proteins, lipids and DNA), as well as the abundances of viruses and that of prokaryotes in the surface seawaters and in the aerosol’s fine (<1.2 µm) or coarse (>1.2 µm) fractions, as determined during bubble-bursting experiments. Reported are average values and standard deviations for the different samples analysed in five experiments over a period of two weeks, from 19 June to 04 July 2006.

In the present study, in order to characterise different organic compounds of marine aerosols we simulated the generation of airborne sea-sprays through bubble-bursting experiments conducted in the North-Eastern Atlantic Ocean offshore Ireland (Fig. 1 and Supplementary Figure 1). The selected area represents a clean oceanic sector^33, 34^, free from the influence of major anthropogenic sources^5, 14^. The experimental production of sea-sprays, as well as investigations of natural aerosols sampled in marine and coastal areas, typically reveals the presence of large fractions of organic compounds and microorganisms of marine origin^5, 11, 20, 22, 28, 35^. Our results are consistent with previous findings, indicating the presence of detectable amounts of organic matter in the experimentally produced aerosols, both in the fine (<1.2 µm) and in coarse (>1.2 µm) fractions (Fig. 2). There is evidence that the sea surface microlayer is enriched in biogenic molecules such as carbohydrates, lipids and proteinaceous material^36^. We show here that the main biochemical classes of organic compounds (i.e., lipids, proteins and carbohydrates) as well as the biopolymeric carbon (as the sum of lipid, protein and carbohydrate carbon content) are transferred from surface seawaters to marine aerosols, along with extracellular DNA, cell fragments, intact viruses and bacteria (Fig. 2).

**Figure 3:**
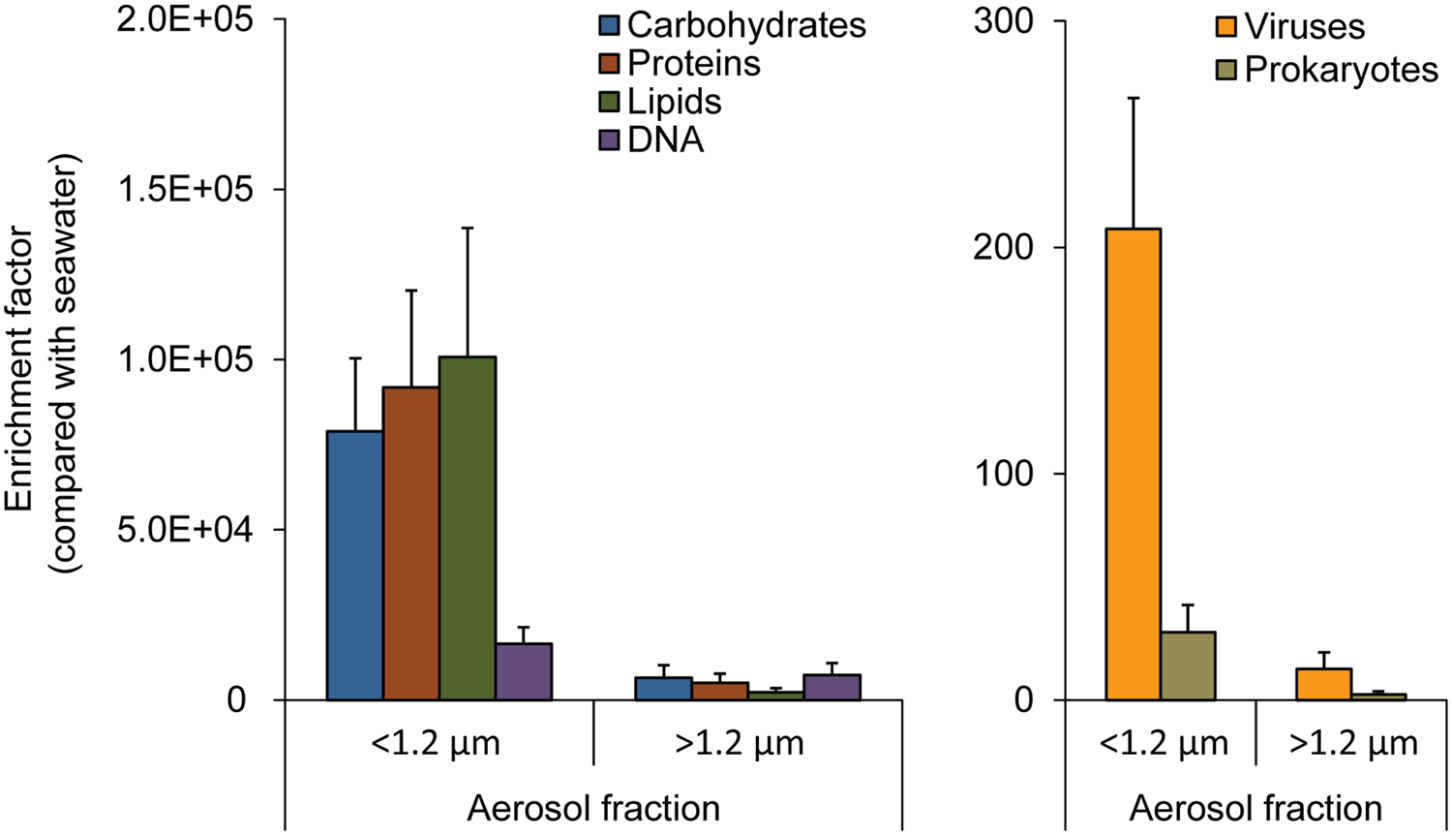
Enrichment in organic matter and microbes in aerosol samples with respect to seawater. The figure reports the enrichment factors (see methods section for calculation) of the different organic compounds analysed in the present study (carbohydrates, proteins, lipids and DNA), as well as of viruses and prokaryotes in fine-sized (<1.2 µm) and coarse-sized (>1.2 µm) aerosol samples. Reported are average values for the two weeks of bubble-bursting experiments, with bars representing standard deviations.

**Figure 4:**
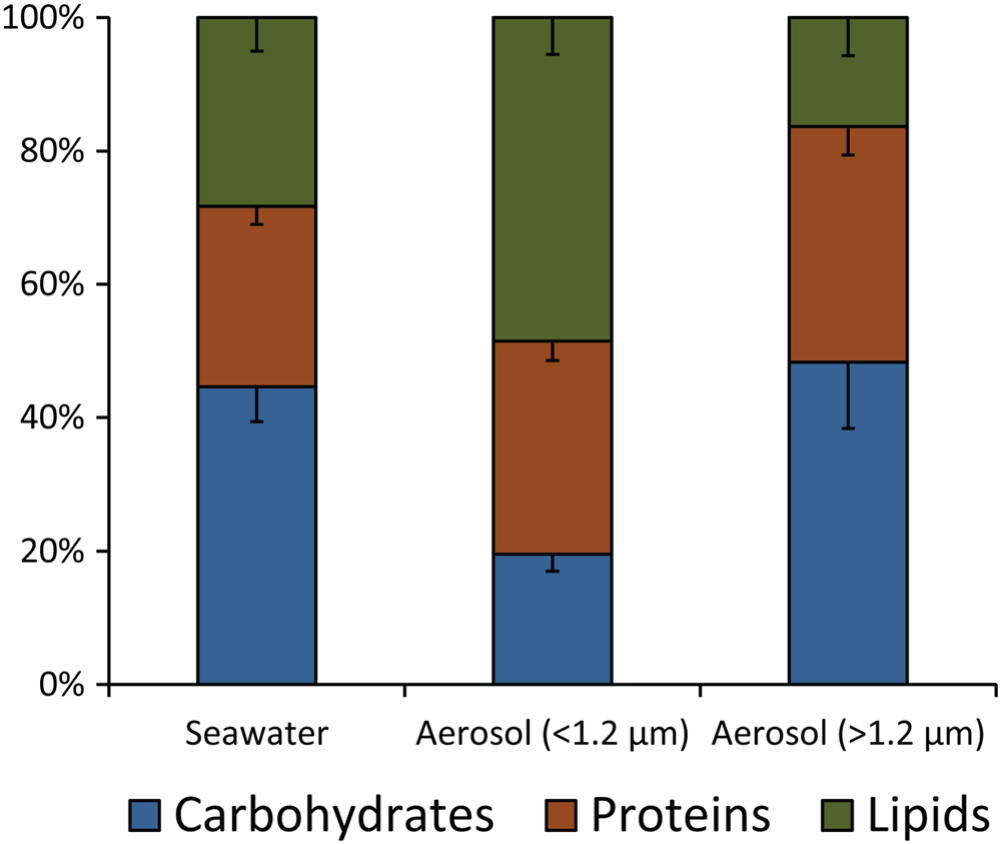
Composition of the biopolymeric carbon in seawater and aerosol samples. The figure shows the relative contribution of carbohydrates, proteins and lipids to the overall biopolymeric carbon contained in the source-seawater and in the fine (<1.2 µm) or coarse (<1.2 µm) aerosol fractions, reporting average values and standard deviations over the two weeks of bubble-bursting experiments.

Our results indicate that the experimentally produced aerosols were highly enriched in all of the organic components analysed when compared with seawater samples (Fig. 3). The enrichment factors, for all the variables, were significantly higher (p < 0.01) in the fine aerosol fractions. We reported the highest enrichments for lipids (up to 140,000x), followed by carbohydrates (up to 100,000x), proteins (up to 120,000x), DNA (up to 30,000x), viruses (up to 250x) and prokaryotic cells (up to 45x) (Fig. 3). The different enrichment factors contributed to the changes in the composition of the total biopolymeric carbon pools observed between seawater and aerosol samples (Fig. 4).

The hydrophobic nature and surface-active properties of lipids may favour their sea-to-air transfer^9, 34, 37^. Our data confirm these expectations that lipids are preferentially transferred^14, 37–39^, possibly also as a result of their association with other compounds favouring co-aerosolization processes^11, 40^. Among the concentrations of the three biochemical classes of organic compounds of the fine aerosol particles, only those of lipids were significantly correlated with those observed in seawater samples (Supplementary Table 1). Microorganisms have the potential to affect cloud formation without leaving the Earth’s surface by releasing hydrophobic biosurfactants (including rhamnolipids, glycolipids and lipopeptides) in the environment and atmospheric aerosols^41^. As such, our results suggest that enhanced biological activity and release of lipid compounds at the ocean surface layer can significantly increase the carbon content of the generated marine aerosols.

The other classes of organic compounds (proteins and carbohydrates) displayed a high enrichment factor (up to 10^5^), but their concentrations in the aerosol did not reflect the patterns observed in seawater samples (Supplementary Table 1). This indicates that factors other than the physical ones reported above for lipids can control the transfer of the different organic compounds from the ocean surface layer to the atmosphere. These can include biological processes driving the properties of the organic matter in surface oceanic waters, such as changes in activity and composition of phytoplankton^42^, viral demise of algal blooms and viral shunt of prokaryotic assemblages^18, 43, 44^, differences in the aggregation properties of the marine organic matter^25, 45^, as well as in the fraction of bacteria attached to suspended particles^24^.

The DNA concentrations in both fine and coarse aerosol fractions reflected those in the seawater samples (Supplementary Table 1). To our knowledge, this is the first study reporting that aerosols can display high enrichment factors for DNA. Assuming a DNA content of 0.09 fg per virus and 3.2 fg DNA per prokaryotic cell^46, 47^, our results indicate that viruses and prokaryotes contributed only for 0.5–6.6% of the total DNA content of the organic marine aerosol, suggesting that most of the DNA was extracellular or of eukaryotic origin. These results lead to hypothesize that processes driving the release of extracellular DNA in oceanic surface waters, such as grazing and viral lysis of eukaryotic and prokaryotic cells^48, 49^, may strongly influence the quantity and composition of organic matter pools of aerosols, especially of their submicron size. However, there are no studies to date on the fate of this biological molecule in the aerosol, nor evidences if aerosolized DNA can drive cloud condensation and/or or ice nucleation, influencing albedo and climate.

The abundance of viruses and prokaryotes in aerosol samples (0.3–3.5 × 10^5^ viruses m^−3^ and 0.1–2.1 × 10^5^ cells m^−3^), were consistent with abundances reported so far for natural marine aerosols (0.2–1.8 × 10^5^ viruses m^−3^ and 0.1–1.2 × 10^5^ cells m^−3^)^20, 28^. These results further support the conclusion that our experimental setup simulated realistically the aerosol production processes occurring in the field^14, 50^. Assuming an average carbon content of 0.2 fg per viral particle and 20 fg per prokaryotic cell^43^, we estimated that viruses and prokaryotes contributed for less than 0.1% of the biopolymeric organic carbon contained in the aerosols. This result further support the conclusion that the labile fraction of organic aerosol fractions can be enriched in cell debris, exudates, colloidal materials and other dissolved forms of organic compounds of phytoplankton origin^21^. Our results also indicate that 15–25% of the total aerosol viruses and 10–20% of total aerosol prokaryotes were exclusively associated to the fine aerosol fraction (<1.2 µm). This suggests that previous investigations on marine organic aerosols, based on 1-µm pore size filters^20^, underestimated the sea-to-air transfer of microbes.

The abundances of prokaryotes in the aerosol and in seawater were tightly coupled (Supplementary Table 1), suggesting that the contribution of prokaryotes to the organic fraction of aerosols can be higher in marine areas displaying higher prokaryotic abundances in surface seawaters. Our results show that in the aerosol viruses were ca. six times more enriched than prokaryotes (Fig. 3 and Supplementary Table 1), suggesting their preferential transfer to the atmosphere, possibly related to their smaller size. Considering that marine aerosols can contain viable bacteria^51–53^ and that they can persist in the atmosphere for weeks being possibly active^54, 55^, our results let to hypothesise the possibility of viral infection of bacteria present in the marine aerosols. The virus-prokaryote interactions would thus represent a biological factor, in addition to the chemical and physical processes, potentially influencing the aerosol’s properties.

**Figure 5:**
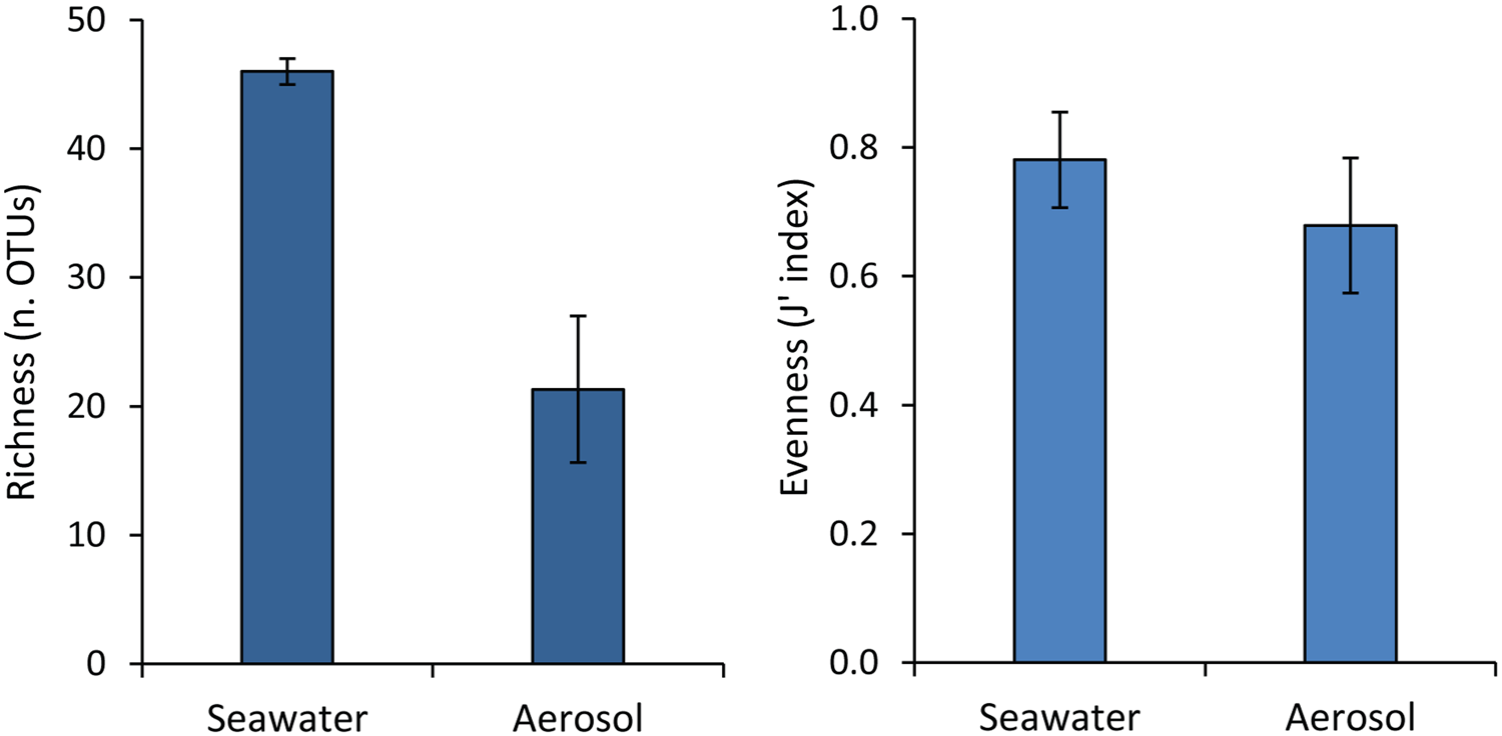
Diversity of bacterial genotypes in surface seawater and aerosol samples. The figure shows the richness (as number of different bacterial OTUs determined by ARISA) and evenness (Pielou Index, J’) of the bacterial genotypes recovered from aerosol samples and from the source-seawaters used for their generation. Reported are average values for the two weeks of bubble-bursting experiments, with bars representing standard deviations.

**Figure 6:**
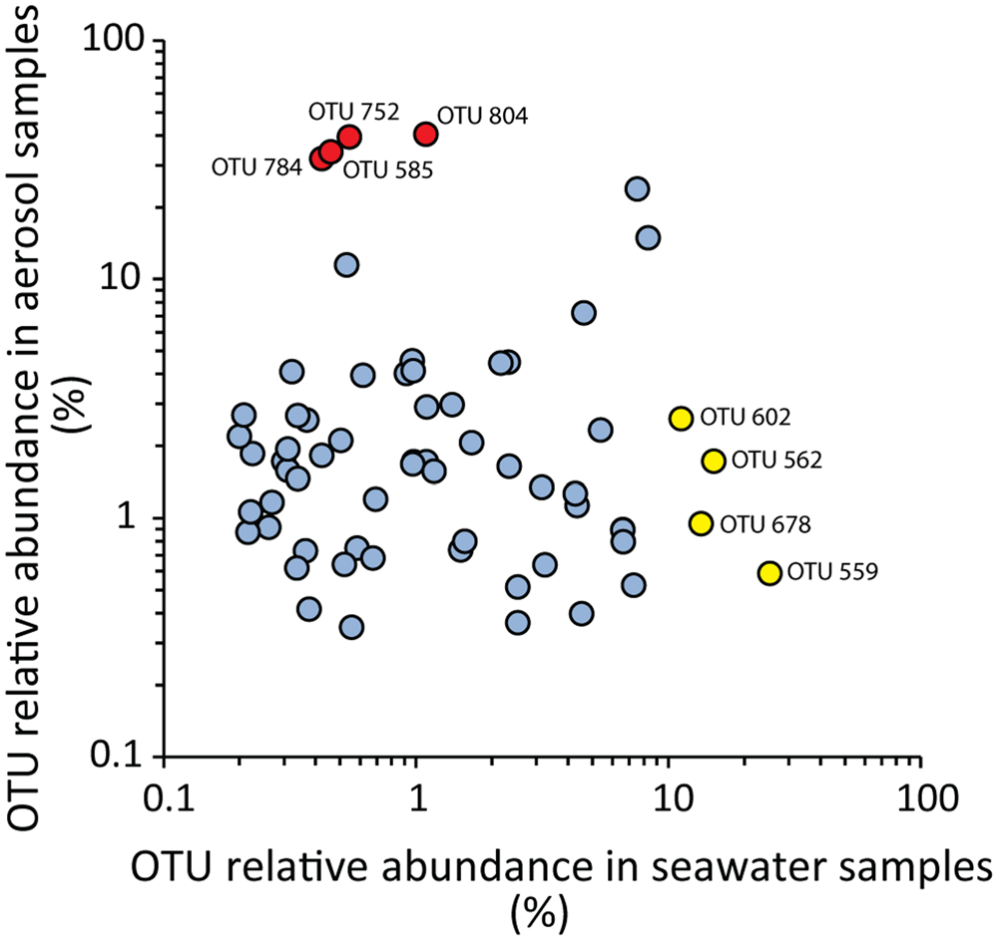
The dominant bacterial OTUs are different in seawater and in aerosol samples. The OTUs shared between seawater and aerosol samples (i.e., those OTUs identified to be subjected to sea-to-air transfer) are shown in the scatterplot. The top-four most abundant OTUs identified in seawater and aerosol samples are highlighted in yellow and in red, respectively. The OTU ID indicates the base pair lengths (see methods section) of the ARISA fragments.

Several studies provided evidence that the sea surface microlayer hosts distinct microbial assemblages involved in key biogeochemical processes and air–sea gas exchange^36, 56^. However, few studies have been conducted to investigate the diversity of prokaryotes in natural marine aerosols^28, 52, 57^, and even less is known on the potential selective transfer of different oceanic bacterial taxa from the sea surface to the aerosol. To this regard, clone libraries of 16 S rRNA gene obtained from seawater samples collected in coastal systems of the Arctic Ocean and experimentally generated aerosols provided some insights into a selective enrichment of different bacterial taxa in the aerosol^58^. Our findings, based on molecular fingerprinting of the bacterial 16 S ribosomal genes carried out on oceanic samples, indicate that the produced aerosols displayed a lower richness of bacterial OTUs when compared to those in the seawater (Fig. 5). In fact, the experimentally produced marine aerosols contained only a fraction of the bacterial genotypes found in the source-seawater (Fig. 6 and Supplementary Figure 2), reinforcing previous findings obtained from coastal marine ecosystems of a preferential transfer of specific bacterial taxa from the ocean surface layer to the aerosol. The bacterial genotypes dominant in seawater were under-represented in the marine aerosols (Fig. 6 and Supplementary Figure 2). Conversely, several less abundant bacterial taxa present in seawater represented the major portion of bacteria transferred to the marine aerosol. Although these findings need to be refined through the identification of the bacterial taxa using high throughput sequencing approaches, a possible explanation of this result could be the different surface properties of bacterial taxa, which in turn can influence their selective transfer from the sea surface to the aerosol^59^.

Recent results have highlighted the large ecological impacts of the aerosol-mediated transfer of microbes^22, 53^. Our results suggest that less abundant bacterial taxa, as a result of their transfer to the marine aerosol, can be spread over wide oceanic regions. The potential of aerosol of representing a factor for the dispersal of these taxa opens important ecological and evolutionary perspectives. One of the pioneer theories of microbial ecology states that: “*everything is everywhere, but the environment selects*”^60^. In this regard, our results suggest that marine aerosol can be included along with marine currents as one of the factors contributing to bacterial dispersal in the oceans. We do not know yet the selective forces controlling bacterial survival in the marine aerosol. However, bacterial adaptation to aerosols’ conditions could facilitate their persistence, representing a key element for the fitness of bacterial taxa in different oceanic regions. Indeed, a successful dispersal via marine aerosols might increase the possibility by bacterial taxa to find new oceanic regions suitable for their growth, escaping from local dominant competitors. This would extend our notion of the recently proposed concept of a persistent global microbial “seed bank” existing in the seawater column and sediments^61, 62^, paving the way for the study of the biological and ecological effects of the sea-to-air transfer and dispersal through aerosols of prokaryotes and viruses on the global distribution and local diversification of marine microbes.

Overall, our study provides novel insights into the quantity, composition and biological factors able to influence the organic fraction of marine aerosols. We provide evidence that the sub-micron aerosol particles can be highly enriched in lipids, proteins, carbohydrates, DNA, prokaryotic cells and viruses of seawater origin. We also show that different organic compounds, as well as different bacterial genotypes, show a preferential sea-to-air transfer. These results open new perspectives for the study of the global relevance of the microbial dispersal via aerosolization and of the mechanisms underlying the observed selective transfer of organic matter and microbes from the ocean surface to the marine aerosol.

## Methods

### Study area and experimental setup

Samples of seawater were collected 400 km off the west coast of Ireland in the North Eastern Atlantic Ocean on board R/V Celtic Explorer (Supplementary Figure 1), to produce primary marine aerosol through bubble bursting experiments as previously detailed^14^. Briefly, airtight high-grade stainless steel bubble bursting tanks (200 litres in volume) were filled with 100 litres of seawater through a water supply system (Fig. 1), with an inlet below the ship bow continuously delivering water from 2 m depth below the sea surface at a flow rate of 6–7 L min^−1^. A bubble-generating system run at seawater flow rate of 20 L min^−1^ was used to produce bubbles, in turn bursting at the water surface forming sea spray aerosol^14^ (Fig. 1). Such a system has been proven to produce bubble size spectra representative of the breaking waves at open sea and to generate sea-spray particle size distributions representative of the natural ones^50^. Aerosol particles were collected over 12 hours from the beginning of the bubble bursting experiments by using five quartz filters (with a pore size ranging from 0.05 to 10 μm) mounted on a five stage Berner impactor operating at a flow rate of 80 L min^−1^. The concentration of aerosol particles in the tank was set to 3.1 ± 0.8 × 10^3^ cm^−3^, leading to an estimated production of 2.5 ± 0.7 × 10^7^ particles m^−2^ s^−1^ to overlap the range of previously observed *in situ* source fluxes^14, 63, 64^. For biochemical, microbiological and molecular analyses, quartz filters were stored at −20 °C until laboratory essay. To provide contextual evaluation of the variables measured in quartz filters, the seawater used for aerosol generation was sampled and pre-filtered onto 10 µm pore-size filters, then processed for biochemical, microbiological and molecular analyses as described below. Overall, we conducted five experiments over a period of two weeks from 19 June to 04 July 2006, and three replicates were analysed in each experiment for each investigated variable. Molecular analyses were conducted only on three of the experiments, because of lack of sufficient material available for analysis in the other two.

### Analyses of carbohydrates, proteins, lipids and DNA in the seawater and marine aerosol

Quartz filters used for collecting aerosol during bubble bursting experiments were homogenised in sterile and virus free MilliQ water (pre-filtered onto 0.02-μm pore-size filters). The homogenates of quartz filters and the polycarbonate filters (Sterlitech, 0.05 µm pore-size) used for concentrating seawater particles were then treated with ultrasounds (three 1-min treatments using Branson 2200 Sonifier, 60 W, 47 kHz) before carbohydrate, protein and lipid determinations carried out using spectrophotometric analyses. Following further tests, we verified that the sonication step initially included was not strictly necessary as it did not influence the results in our specific case, thus it can be omitted in similar analyses in the future (Supplementary Figure 3). Carbohydrate analysis was carried out using the widely applied phenol–sulfuric acid method^65^. This method is based on dehydration of hydrolyzed saccharides to furfural derivatives during reaction with concentrated sulphuric acid. Furfural derivatives with phenol forms colored complexes that absorb light in the visible range, with a maximum absorbance at wavelength of 490 nm. Protein analysis was carried out using the Coomassie blue method^66^, properly adapted for the quantification of low protein concentrations^67, 68^. Lipid extraction was carried out by direct elution with chloroform and methanol (1:2 v:v)^69^. The extracted lipids were then dried at 80 °C and determined spectrophotometrically (at wavelength of 375 nm) after carbonisation at 200 °C in concentrated sulphuric acid^70^.

Carbohydrate, protein, and lipid concentrations in seawater and aerosol samples were obtained by calibration curves using standard solutions of glucose (from 1 to 25 μg ml^−1^), bovine serum albumin (from 0.5 to 10 μg ml^−1^), tripalmitin (from 2.5 to 25 μg ml^−1^), respectively. For all analyses clean filters of the different pore sizes were processed according to the procedures described above and used as blanks. The detection limits determined through the analysis of blanks added with internal standards were 2.5 μg ml^−1^, 1.0 μg ml^−1^ and 5 μg ml^−1^ for carbohydrates, proteins and lipids, respectively.

The analyses of DNA in aerosol and seawater samples were performed fluorometrically using SYBR Green I as fluorochrome after digestion of RNA with RNase (DNase free, 1 U ml^−1^ for 15 minutes at room temperature)^71^. DNA concentrations were obtained by calibration curves using standard solutions of DNA from *E. coli* (1–100 ng ml^−1^).

Clean quartz and polycarbonate filters were used as blanks. Carbohydrate, protein, lipid and DNA concentrations obtained from the analyses of 0.05–1.2 μm pore-size quartz filters were summed and defined as belonging to the fine aerosol fraction (<1.2 μm) of the aerosol, whereas concentrations obtained from the analyses of 1.2–10 μm pore-size quartz filters were defined as belonging to the coarse aerosol fraction (>1.2 μm).

### Analyses of prokaryotic and viral abundance in the seawater and marine aerosol

Seawater samples were filtered onto Anodisc 25 mm (20 nm pore size) filters. To determine viral and prokaryotic abundance in the aerosol, each size class quartz filter was homogenised in sterile and virus free MilliQ water (pre-filtered onto 0.02 μm pore-size filters) and samples were then treated by ultrasounds (three 1-min treatments using a Branson Sonifier 2200, 60 W 47 kHz) and pyrophosphate (5 mM final concentration) to detach viruses and prokaryotes. Aerosol samples were then centrifuged at 800 × g for 10 minutes and the supernatant filtered onto Anodisc 25 mm (20 nm pore size) filters. All Anodisc filters were stained with 20 μl of SYBR Green I (diluted 20 fold in MilliQ water) for 15 minutes in the dark and then rinsed twice with 1 ml MilliQ water in order to eliminate fluorescence background noise. The filters were mounted on slides using an anti-fade solution (50% phosphate buffer, 6.7 mM, pH 7.8; 50% glycerol containing 0.25% ascorbic acid) and at least 100 optical fields at 1000 × magnification were examined under epifluorescence microscopy using a Zeiss Axioskop 2 Mot microscope, equipped with a 100 W lamp.

### Intracellular DNA recovery for molecular analysis

The prokaryotic cells contained in the 10-μm pre-filtered source-seawater were concentrated onto 0.05 µm pore size polycarbonate filters (Sterlitech). The corresponding aerosol samples generated during the bubble-bursting experiment were obtained by pooling all quartz filters of the different pore sizes (0.05–10 μm). The DNA contained in the prokaryotic cells collected on the filters was extracted and purified using the QIAamp DNA Micro Kit (QIAGEN). This kit was preferred based on preliminary tests of DNA extractions from quartz filters, indicating higher DNA yields than with the MoBio UltraClean Microbial DNA isolation kit (Supplementary Figure 4). Before DNA extraction, the seawater and aerosol samples were treated with DNAse I (5U ml^−1^) to remove any possible extracellular DNA contamination^71^. The purified extracts of intracellular DNA were then analysed by molecular fingerprinting (through ARISA - Automated Ribosomal Intergenic Spacer Analysis)^72^ to provide information on the bacterial diversity in seawater and in the corresponding produced aerosols.

For ARISA, the purified intracellular DNA was amplified using universal bacterial primers 16S-1392F (5′-GYACACACCGCCCGT-3′) and 23S-125R (5′-GGGTTBCCCCATTCRG-3′). This allowed the amplification of the ITS1 region in the rRNA operon plus ~282 bases of the 16 S and 23 S rRNA^72^. Primer 23S-125R was fluorescently labelled with the fluorochrome HEX (MWGspa BIOTECH). PCR reactions were performed in 50 µl volumes in a thermalcycler (Biometra) using the MasterTaq^®^ kit (Eppendorf), which reduces the effects of PCR-inhibiting contaminants. We used 30 PCR-cycles, consisting of 94 °C for 1 minute, 55 °C for 1 minute and 72 °C for 2 minute, preceded by 3 minutes of denaturation at 94 °C and followed by a final extension of 10 minutes at 72 °C. To check for eventual contamination of the filters, consumables and PCR reagents, negative controls of MoBio extracts from blank filters (i.e., control filters with no seawater or aerosol sample) containing the PCR-reaction mixture were run during each PCR reaction. All the negative controls produced no ARISA amplicons, confirming the lack of contamination and the high confidence of the analytical approach. Positive controls containing genomic DNA of *E. coli* were used and PCR amplicons were checked on agarose-TBE gel (1%), containing ethidium bromide for DNA staining and visualization. Four different reactions were run for each sample and then combined to form two duplicates, subsequently utilised for independent ARISA reactions. The two resulting PCR combined products were purified using the Wizard PCR clean-up system (Promega, Madison, Wis), resuspended in 50 µl of MilliQ water supplied with the clean-up system and then quantified spectrofluorimetrically as described above. For each ARISA run, 5 ng of purified amplicons were mixed with 14 µl of internal size standard (GS2500-ROX; Applied Biosystems, Foster City, Calif.) in deionised formamide, then denatured at 94 °C for 2 minutes and immediately chilled in ice. Automated detection of ARISA fragments were carried out using ABI Prism 3100 Genetic Analyzer (Applied Biosystems). ARISA fragments were determined using Peakscanner analytical software (ABI) and the results analysed using standardization of fluorescence among samples, elimination of “shoulder” and non-replicated peaks, and cut-off criterion^73^. Bacterial genotype richness was expressed as the total number of peaks within each electropherogram, while the evenness (Pielou index, J’) was calculated in order to assess the relative importance of each taxon within the entire assemblage.

### Calculation of Enrichment factors

Measures on surface-water and contextually produced bubble bursting aerosol were compared by means of enrichment factors, following the equation:

$$EF=\frac{{(\frac{X}{Na})}_{aerosol}}{{(\frac{X}{Na})}_{seawater}}$$

Where EF is the enrichment factor for the X variable and Na is sodium concentration (Supplementary Figure 5), used as mass indicator for data normalization^74, 75^.

## Electronic supplementary material

Supplementary Information
