# Supplementary material for: Transfer of labile organic matter and microbes from the ocean surface to the marine aerosol: an experimental approach
Source: Sci Rep. 2017 Sep 13;7:11475. doi: 10.1038/s41598-017-10563-z (PMC5597575; doi:10.1038/s41598-017-10563-z)
Supplement: Supplementary file 1 — Supplementary Information [file 41598_2017_10563_MOESM1_ESM.doc]

**Supplementary Information**

**Transfer of labile organic matter and microbes from the ocean surface to the marine aerosol: an experimental approach**

Eugenio Rastelli^1,2^, Cinzia Corinaldesi^3^, Antonio Dell’Anno^1^, Marco Lo Martire^1^, Silvestro Greco^4^, Maria Cristina Facchini^5^, Matteo Rinaldi^5^, Colin O’Dowd^6^, Darius Ceburnis^6^, Roberto Danovaro^1,2*^

^1^Department of Life and Environmental Sciences, Polytechnic University of Marche, Ancona, Italy

^2^Stazione Zoologica Anton Dohrn, Villa Comunale, Naples, Italy

^3^Department of Sciences and Engineering of Materials, Environment and Urbanistics, Polytechnic University of Marche, Ancona, Italy

*^4^Istituto Superiore per la Ricerca Ambientale, ISPRA, Roma, Italy*

*^5^Institute of Atmospheric Sciences and Climate (ISAC), National Research Council (CNR), Bologna, Italy*

*^6^School of Physics & Centre for Climate and Air Pollution Studies, Ryan Institute, National University of Ireland, Galway, Galway, Ireland.*

***Corresponding author**: Roberto Danovaro

Department of Life and Environmental Sciences

Università Politecnica delle Marche

Via Brecce Bianche, 60131, Ancona, Italy

Tel: +39-071-2204654 - Fax: +39-071-2204650

E-mail: [r.danovaro@univpm.it](mailto:r.danovaro@univpm.it)

**This file includes:**

**Supplementary Table 1**

**Supplementary Figures 1-5**

**Supplementary Table 1. Correlation analyses between the concentration of different organic matter compounds and microbes in surface seawater and aerosol samples.** The table reports the results of the correlation analyses between the contents of carbohydrates, proteins, lipids, DNA, viruses and prokaryotes determined in seawater and those measured in the aerosol’s fine (<1.2 µm) or coarse (>1.2 µm) fractions. Reported are the number of observations (n), the coefficients of the correlation (r) and level of significance at p<0.05 (*) and p<0.01 (**). n.s.= not significant

|  |  | n | r | *p* |
| --- | --- | --- | --- | --- |
| Carbohydrates | Seawater vs Aerosol (fine fraction) | 15 | 0.417 | n.s. |
|  | Seawater vs Aerosol (coarse fraction) | 15 | -0.08 | n.s. |
| Proteins | Seawater vs Aerosol (fine fraction) | 15 | 0.419 | n.s. |
|  | Seawater vs Aerosol (coarse fraction) | 15 | -0.239 | n.s. |
| Lipids | Seawater vs Aerosol (fine fraction) | 15 | 0.972 | ****** |
|  | Seawater vs Aerosol (coarse fraction) | 15 | -0.299 | n.s. |
| DNA | Seawater vs Aerosol (fine fraction) | 15 | 0.737 | ****** |
|  | Seawater vs Aerosol (coarse fraction) | 15 | 0.736 | ****** |
| Viruses | Seawater vs Aerosol (fine fraction) | 15 | 0.113 | n.s. |
|  | Seawater vs Aerosol (coarse fraction) | 15 | 0.542 | ***** |
| Prokaryotes | Seawater vs Aerosol (fine fraction) | 15 | 0.992 | ****** |
|  | Seawater vs Aerosol (coarse fraction) | 15 | 0.852 | ****** |

**
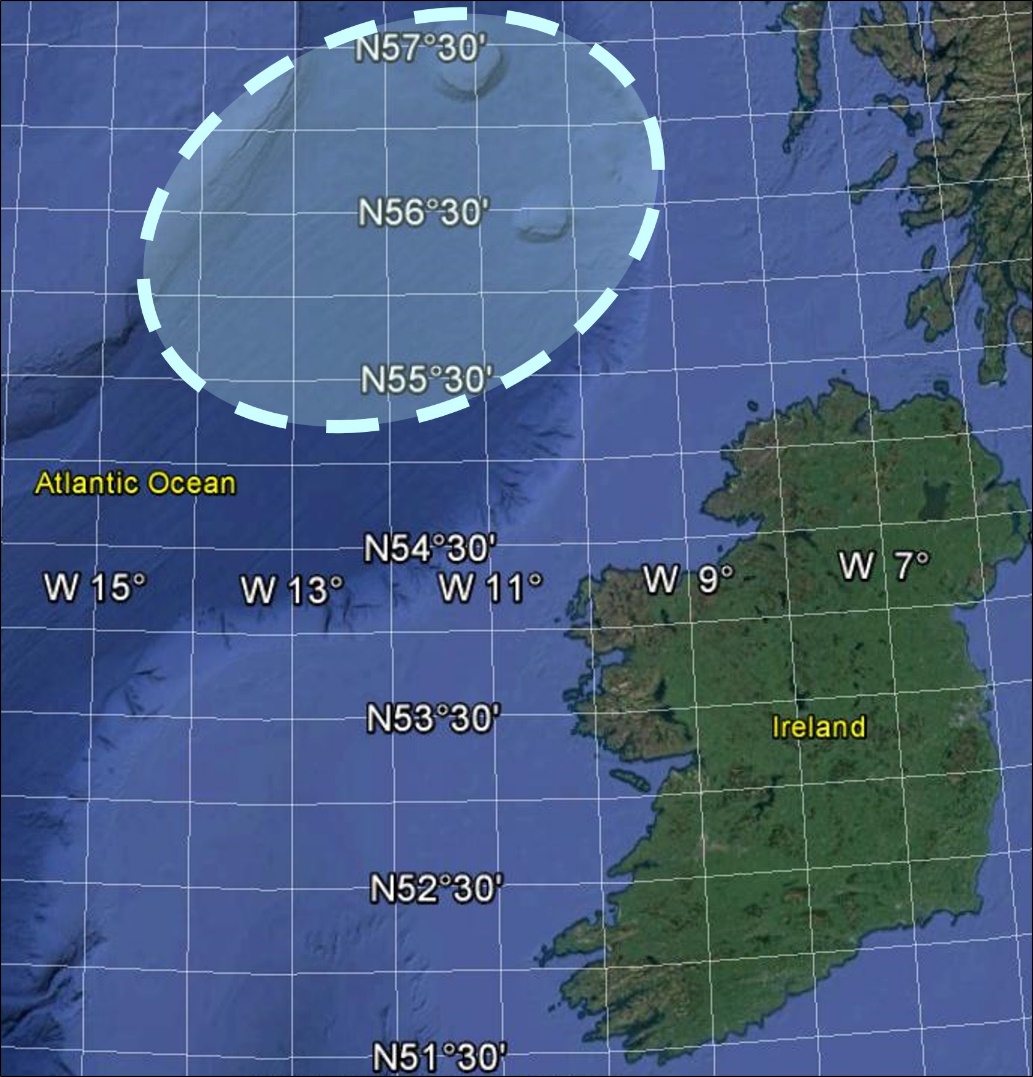
**

Data SIO, NOAA, U.S. Navy, NGA, GEBCO

Image Landsat/Copernicus

Google Earth

**Supplementary Figure 1. Sampling site.** The map shows the location of the clean oceanic sector (enclosed by the light-blue ellipse in the figure, generated using Google Earth, version 7.1.7.2606, available at the following URL: https://www.google.com/earth/) in which bubble-bursting experiments were conducted during oceanographic cruises conducted onboard R/V Celtic Explorer.

**Supplementary Figure 2. Bacterial assemblage composition in surface seawater and aerosol samples**. The heatmap shows the relative proportions of the different bacterial OTUs identified in aerosol samples and in the source-seawaters during three bubble-bursting experiments conducted on 19 June, 28 June and 2 July. The same OTUs highlighted in Figure 6 (see main text) as the top-four most abundant in seawater and aerosol samples, are highlighted here in yellow and in red, respectively. The OTU IDs reported indicate the base pair lengths of the ARISA fragments. SW, seawater samples used for aerosol generation; Aer, aerosol samples.


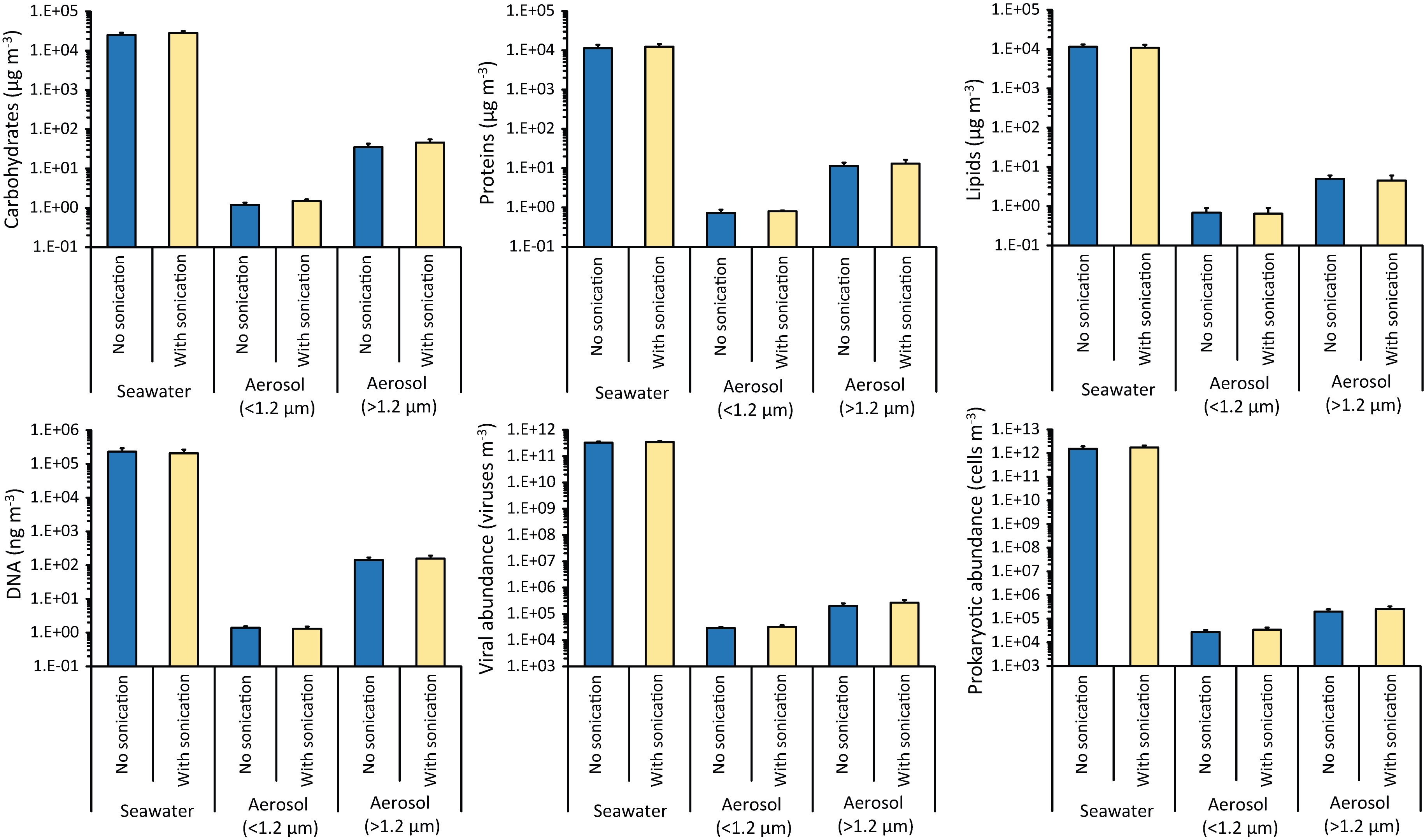


**Supplementary Figure 3. Testing the effect of sonication on the analysis of organic matter and microbes in surface seawater and aerosol samples.** The panel reports the concentrations of the different organic compounds analysed in the present study (carbohydrates, proteins, lipids and DNA), as well as the abundances of viruses and that of prokaryotes in the surface seawaters and in the aerosol’s fine (<1.2 µm) or coarse (>1.2 µm) fractions. Compared are results based on the application of the sonication step (blu bars) or not (yellow bars). Aliquots from samples of the 19 June were used in these tests, showing no effects of the sonication step. Reported are average values and standard deviations for the samples analysed.

**
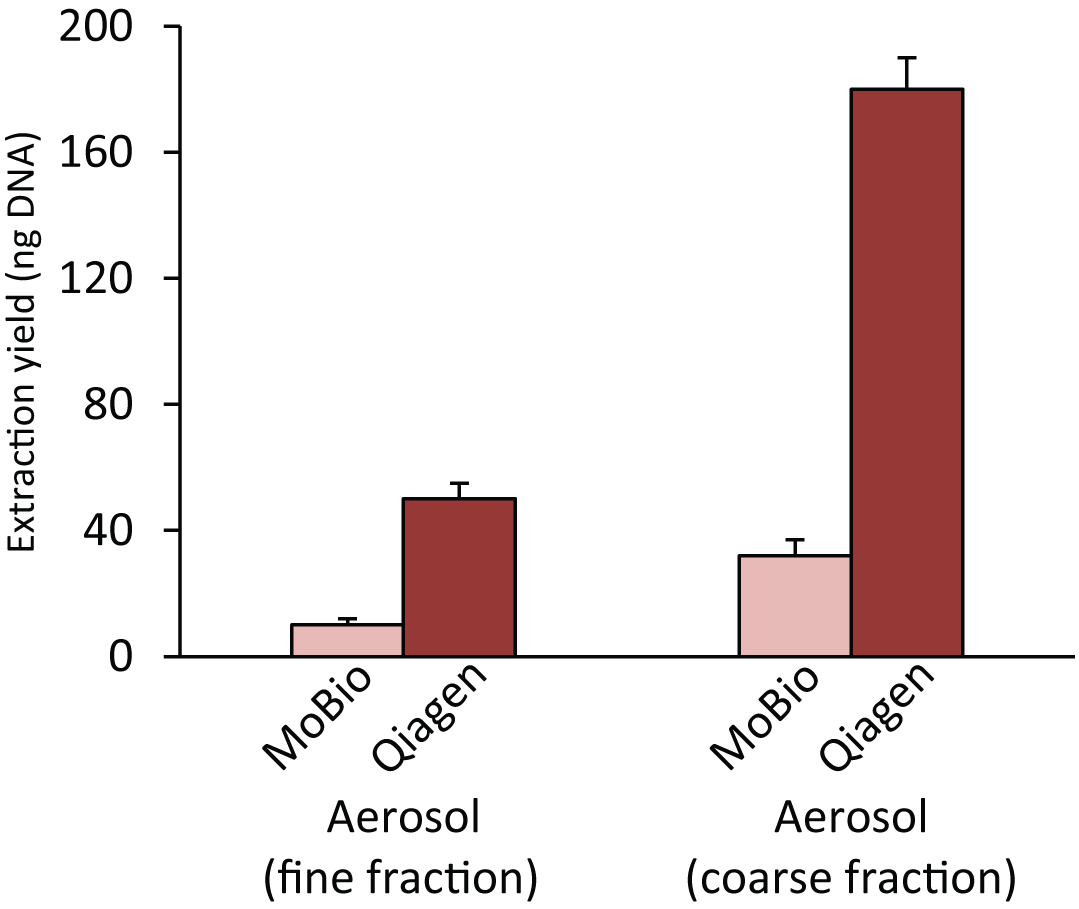
**

**Supplementary Figure 4. DNA yields using different commercial kits.** The figure reports the comparison between the extraction efficiency of two commercial kits (MoBio-UltraClean Microbial DNA isolation Kit and Qiagen-QIAamp DNA Micro Kit). Reported are average values and standard deviations for triplicate DNA extraction reactions from aerosol filters of the fine (<1.2 µm) and coarse (>1.2 µm) fractions.

**
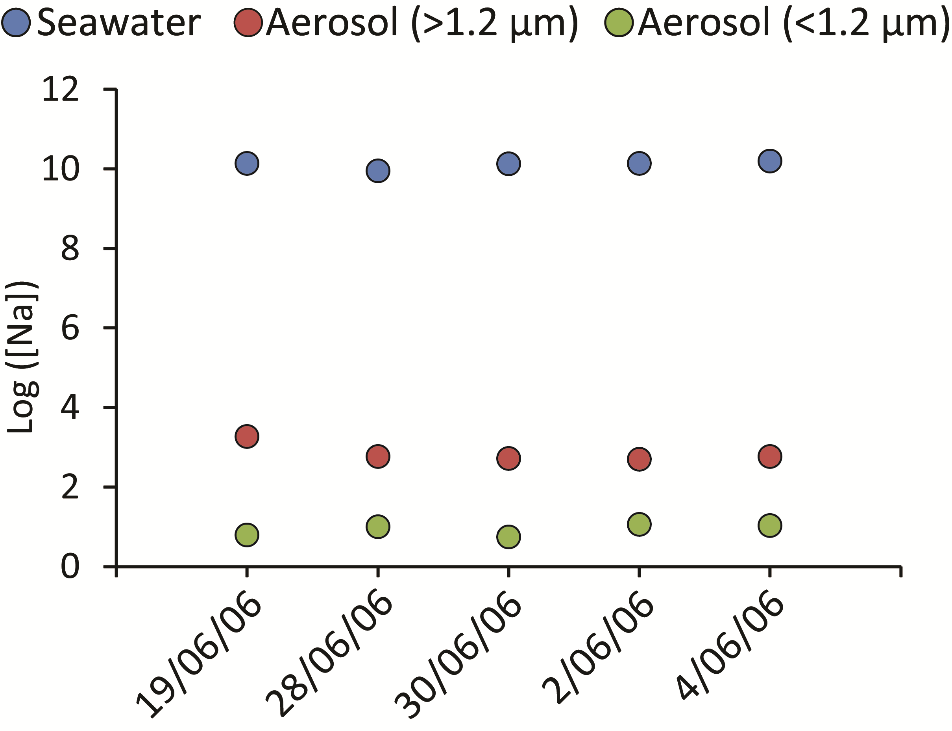
**

**Supplementary Figure 5. Concentrations of sodium in seawater and aerosol.** The figure reports the amount of sodium (Na), expressed as log_10_ of the concentrations of Na^+^ (in µg Na^+^ m^-3^), in the source-seawater samples used in the different bubble bursting experiments, as well as in the fine (<1.2 µm) and coarse (>1.2 µm) fractions of the corresponding aerosol samples generated.
